# Subterranean termite phylogeography reveals multiple postglacial colonization events in southwestern Europe

**DOI:** 10.1002/ece3.2333

**Published:** 2016-07-27

**Authors:** Thomas Lefebvre, Edward L. Vargo, Marie Zimmermann, Simon Dupont, Magdalena Kutnik, Anne‐Geneviève Bagnères

**Affiliations:** ^1^ Institut de Recherche sur la Biologie de l'InsecteUMR CNRS 7261UFR Sciences et TechniquesUniversité François Rabelais37200ToursFrance; ^2^ Department of Entomology2143 TAMUTexas A&M UniversityCollege StationTexas77843‐2143USA; ^3^ FCBA – Institut technologiqueDpt Biologie et Préservation du BoisAllée de Boutaut BP22733000BordeauxFrance; ^4^ Present address: YnsectR&D dptGenopole 391058EvryFrance

**Keywords:** Iberian Peninsula, Messinian salt crisis, Miocene, Morocco, Pleistocene, *Reticulitermes* termites

## Abstract

A long‐standing goal of evolutionary biology is to understand how paleoclimatic and geological events shape the geographical distribution and genetic structure within and among species. Using a diverse set of markers (cuticular hydrocarbons, mitochondrial and nuclear gene sequences, microsatellite loci), we studied *Reticulitermes grassei* and *R. banyulensis,* two closely related termite species in southwestern Europe. We sought to clarify the current genetic structure of populations that formed following postglacial dispersal from refugia in southern Spain and characterize the gene flow between the two lineages over the last several million years. Each marker type separately provided a fragmented picture of the evolutionary history at different timescales. Chemical analyses of cuticular hydrocarbons and phylogenetic analyses of mitochondrial and nuclear genes showed clear separation between the species, suggesting they diverged following vicariance events in the Late Miocene. However, the presence of intermediate chemical profiles and mtDNA introgression in some Spanish colonies suggests ongoing gene flow. The current genetic structure of Iberian populations is consistent with alternating isolation and dispersal events during Quaternary glacial periods. Analyses of population genetic structure revealed postglacial colonization routes from southern Spain to France, where populations underwent strong genetic bottlenecks after traversing the Pyrenees resulting in parapatric speciation.

## Introduction

The Mediterranean basin, particularly the Iberian Peninsula, is one of the most biologically diverse areas in the world (Blondel and Aronson 1999; Médail and Quézel 1999; Myers et al. 2000; García‐Barros et al. 2002; Feliner 2014). In recent years, several studies have focused on the phylogeography and population genetic structures of many Iberian species. Due to the presence of inhospitable habitats and mountain barriers that limit gene flow, several areas of historical endemism have been identified for many species (Cooper et al. 1995; Bilton et al. 1998; Pitra et al. 2000; Llorente et al. 2012; Fernández et al. 2013; Gonçalves et al. 2015). This rich endemism can be explained by the heterogeneous topology of the Iberian Peninsula (it is situated at the interface between the Atlantic Ocean and the Mediterranean Sea), its past connections with Africa (Jaeger 1994; Agustí et al. 2006; Feliner 2014; Husemann et al. 2014), and its role as one of the main glacial refugia in southwestern Europe (Taberlet and Cheddadi 2002).

Indeed, during multiple glacial periods, many plant and animal species persisted on the Iberian Peninsula and subsequently spread and diversified when the climate changed (Paulo et al. 2002; Gómez and Lunt 2007; Rodríguez‐Sánchez et al. 2010; Abellán and Svenning 2014). The distribution and population genetic structure of these species were likely heavily influenced by Quaternary climatic oscillations (Knowles 2001; Hewitt 2004). Most species probably experienced range contractions when temperatures decreased, remaining only in various refugia, and then recolonized ice‐free regions when temperatures increased (Hewitt 1996), or, on the contrary, cold‐adapted species moved to northern ecological niches (Ursenbacher et al. 2015). Such repeated cycles of isolation and dispersal may have driven speciation, through either allopatric adaptation to new environments or the occupation of new niches after sympatric recolonization of deserted areas (Orr and Smith 1998; Hewitt 2001, 2011). Thus, to understand the influence of the glaciation cycles on speciation, studies of subspecies, subpopulations, and recently diverged sister taxa rather than well‐differentiated species may provide greater insight into the processes that led to population differentiation and, ultimately, speciation. Subterranean termites in the genus *Reticulitermes*, with two closely related lineages only recently recognized as separate species, offer an excellent opportunity to investigate the effects of glaciation on colonization and speciation on the Iberian Peninsula.

Using the known ecology of Iberian *Reticulitermes* termites, as well as previous genetic findings (Kutnik et al. 2004; DeHeer et al. 2005; Vargo et al. 2013), it is possible to test some hypotheses regarding the processes responsible for shaping genetic variation within populations in this area. Just like the Italian and Balkan Peninsulas (Uva et al. 2004; Luchetti et al. 2007; Lefebvre et al. 2008; Velona et al. 2010), the Iberian Peninsula served as an ice age refugium and was the evolutionary cradle for two termite lineages, *R. grassei* and *R. banyulensis*, described by Clément et al. (2001) and originally considered subspecies of *R. lucifugus*. After moving northward during a postglacial colonization event, they are now spread over the Iberian Peninsula and southern France, except in the coldest mountains and driest regions (e.g., the Tabernas Desert, Almeria). Their population genetic structure might also be impacted by human‐mediated introductions at much shorter timescales (Dronnet et al. 2005; Lefebvre et al. 2008; Perdereau et al. 2010).

In previous studies, we examined the phylogenetic relatedness of these two Iberian *Reticulitermes* lineages, which differ chemically in their cuticular profiles and morphologically in their postclypei (Clément et al. 2001; Kutnik 2004; Kutnik et al. 2004). Because it is well known that cuticular compounds play a major role in recognition processes between species, their use as markers can be informative in understanding differentiation between lineages. However, many questions remain concerning the phylogeography of these two lineages, especially with regard to the status of some colonies with intermediate chemical profiles or the discrepancies between mitochondrial and nuclear phylogenies.

In this study, we examined the complex evolutionary history of Iberian *Reticulitermes* lineages in light of recently published data on the genus, including estimates of the divergence time between the lineages, combining this new information with more extensive analyses of mitochondrial and nuclear sequence data. To characterize the differentiation among populations distributed over the entire range of *Reticulitermes* in Spain southern France and new samples from northern Africa, we employed comparisons of cuticular hydrocarbons, mitochondrial COI and COII sequences, ITS2 nuclear sequences, and distributions of microsatellite alleles. The combination of multiple markers, each with different evolutionary rates, sheds new light on the current population structure of the Iberian termites and their postglacial dispersal patterns over the last several million years.

## Materials and Methods

### Sample collection

**Figure 1:**
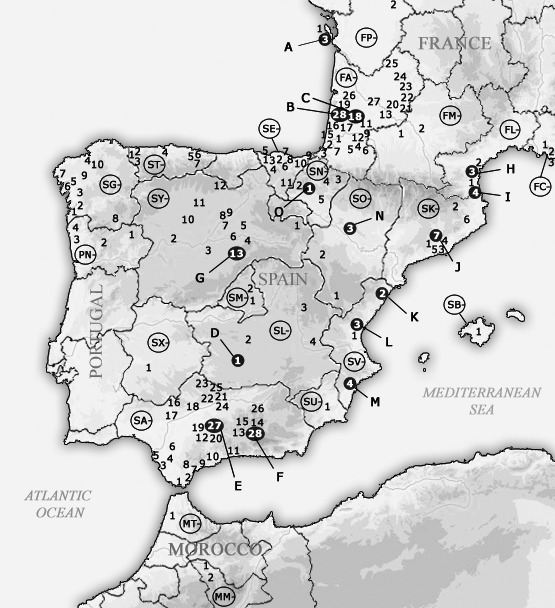
Map showing the location of the sampling sites throughout southwestern Europe and Morocco. Each site is encoded by a number preceded by two letters that correspond to the sampling region (the first letter refers to the country and the second to the administrative region, e.g., SA1 for Spain > Andalusia > locality 1). All localities and their corresponding codes are provided in Table 1. Some sampling sites are labeled with black circles and letters from A to O; they refer to locations where more extensive sampling was conducted to characterize population genetics.

A total of 146 natural and urban sites (coded using two letters that correspond to the country/region of origin and one number that corresponds to the more specific locality; see Table 1 and Fig. 1) were sampled across France, Spain, Portugal, and Morocco. The natural locations were either pine forests or mixed forests, in which termites could be found feeding upon wood debris. In urban locations, termites were collected from damaged wooden structures or bait stations (DOW AgroSciences SAS, Valbonne, France). In nearly all cases (except at several urban locations), at least 20 workers were collected; samples from 98 sites were used for chemical analysis and were placed directly into pentane. Many of these samples could not be used in the genetic analyses because of severe tissue degradation, leaving 39 samples for which we obtained both cuticular hydrocarbon and genetic data.

**Table 1:** Locations where samples were collected, along with the genetic marker(s) and codes used in the analyses. S for Spain, F for France, and M for Morocco. See Figure 1 for location coding. The results of the different analyses are summarized in this table. See also Figure 5A for the spatial relationships of the samples

Code	Locality (Country, Region)	HCs	ITS2	COI ‐ COII	Microsat	Code	Locality (Country, Region)	HCs	ITS2	COI ‐ COII	Microsat
SA1	Tarifa (Spa., Andalucía)		N‐ 1	M‐ 1		SG8	Devesa (Spa., Galicia)			M‐1	
SA2	Sotogrande (Spa., Andalucía)	H‐1		M‐1		SG9	Stgo de Compostela (Spa., Galicia)	H‐1			
SA3	Conil de la Frontera (Spa., Andalucía)		N‐1			SG10	Presedo (Spa., Galicia)	H‐1			
SA4	Medina Sidonia (Spa., Andalucía)		N‐1			ST1	Seares (Spa., Asturias)	H‐2	N‐1		
SA5	Chipiona (Spa., Andalucía)		N‐1			ST2	Seares (Spa., Asturias)	H‐2			
SA6	Jimena de la Frontera (Spa., Andalucía)	H‐1				ST3	Medredos (Spa., Asturias)	H‐2		M‐1	
SA7	Casares (Spa., Andalucía)	H‐1		M‐1		ST4	Villafria (Spa., Asturias)	H‐2	N‐1		
SA8	El Comenar (Spa., Andalucía)	H‐1	N‐1			ST5	Fios (Spa., Asturias)	H‐2	N‐1	M‐1	
SA9	Malaga (Spa., Andalucía)	H‐1				ST6	Fios (Spa., Asturias)	H‐2			
SA10	Mijas (Spa., Andalucía)	H‐1	N‐1			ST7	Fios (Spa., Asturias)	H‐2			
SA11	Salares (Spa., Andalucía)	H‐1	N‐1	M‐2b		SE1	Arborteretza (Spa., Euskadi)	H‐2	N‐1	M‐1	
SA12	Antequera (Spa., Andalucía)		N‐1	M‐2b		SE2	Guernica (Spa., Euskadi)			M‐1	
SA13	Canales (Spa., Andalucía)		N‐1	M‐1		SE3	Bilbao (Spa., Euskadi)	H‐2			
SA14	La Peza (Spa., Andalucía)			M‐1		SE4	Llodio (Spa., Euskadi)	H‐2			
SA15	El Molinillo (Spa., Andalucía)	H‐1		M‐1		SE5	Por.ete (Spa., Euskadi)	H‐2			
SA16	El Pedroso (Spa., Andalucía)	H‐1	N‐1	M‐1		SE6	Amorebieta (Spa., Euskadi)	H‐2			
SA17	Villanueva del Rio (Spa., Andalucía)	H‐1				SE7	Ereño (Spa., Euskadi)	H‐2			
SA18	Lora del Rio (Spa., Andalucía)			M‐1		SE8	Lekeitio (Spa., Euskadi)		N‐1	M‐1	
SA19	Palenciana (Spa., Andalucía)	H‐1	N‐1			SE9	Fuenterrabia (Spa., Euskadi)	H‐1			
SA20	El Tejar (Spa., Andalucía)	H‐1				SE10	Goronaeta (Spa., Euskadi)		N‐1		
SA21	Rabanales (Spa., Andalucía)		N‐1			SE11	Sta Cruz de Campezo (Spa., Navarra)	H‐3			
SA22	Cerro Muriano (Spa., Andalucía)		N‐1	M‐1		SK1	Montblanc (Spa., Cataluña)	H‐3			
SA23	Adamuz (Spa., Andalucía)		N‐1	M‐1		SK2	Castellfollit (Spa., Cataluña)	H‐3			
SA24	Montoro (Spa., Andalucía)			M‐1		SK3	Barcelona (Spa., Cataluña)	H‐3			
SA25	Cardena (Spa., Andalucía)	H‐1	N‐1			SK4	Valldoreix (Spa., Cataluña)	H‐3			
SA26	Belmez (Spa., Andalucía)		N‐1	M‐2a		SK5	SantBoi (Spa., Cataluña)	H‐3		M‐2c	
SA27 /E	Lucena (Spa., Andalucía)		N‐1	M‐2b	S‐3	SK6	Cruïlles (Spa., Cataluña)	H‐3		M‐2c	
SA28 /F	Guadix (Spa., Andalucía)		N‐1	M‐1	S‐2	SK7 /J	Igualada (Fra., Cataluña)		N‐2a	M‐2a	S‐4
SU1	Vélez Rubio (Spa., Murcia)	H‐1				FA1	Peyrehorade (Fra., Aquitaine)	H‐1			
SX1	Badajoz (Spa., Extremadura)	H‐1				FA2	Bayone (Fra., Aquitaine)		N‐1		
SL1 /D	Ciudad Real (Spa., Castilla La Mancha)	H‐1	N‐1	M‐1	S‐2	FA3	St‐Pée‐sur‐Nivelle (Fra., Aquitaine)			M‐1	
SL2	Toledo (Spa., Castilla La Mancha)	H‐1				FA4	Domezain (Fra., Aquitaine)			M‐1	
SL3	Cuenca (Spa., Castilla La Mancha)	H‐1				FA5	Abitain (Fra., Aquitaine)	H‐1			
SL4	Casas Ibanes (Spa., Castilla La Mancha)	H‐1		M‐2a		FA6	Artix (Fra., Aquitaine)	H‐1		M‐1	
SM1	Madrid (Spa., Madrid)	H‐1				FA7	Ayherre (Fra., Aquitaine)			M‐1	
SM2	El Escorial (Spa., Madrid)	H‐1				FA8	Bidache (Fra., Aquitaine)	H‐1			
SO1	Mas de Jacinto (Spa., Aragon)	H‐1	N‐2b	M‐2a		FA9	Grenade‐sur‐Adour (Fra., Aquitaine)	H‐1			
SO2	Campillo (Spa., Aragon)	H‐3				FA10	Grenade‐sur‐Adour (Fra., Aquitaine)	H‐1			
SO3 /N	Zuera (Spa., Aragon)		N‐2a	M‐2c	S‐5	FA11	St Cricq Chaliesse (Fra., Aquitaine)	H‐1			
SV1	Valencia (Spa., Valencia)	H‐1				FA12	Morganx (Fra., Aquitaine)	H‐1			
SV2 /K	Sant Mateu (Spa., Valencia)		N‐2a	M‐2c	S‐5	FA13	Lubbon (Fra., Aquitaine)	H‐1			
SV3 /L	Segorbe (Spa., Valencia)		N‐2b	M‐2a	S‐6	FA14	Le Passage (Fra., Aquitaine)	H‐1			
SV4 /M	Alicante (Spa., Valencia)		N‐2b	M‐2a	S‐6	FA15	Ondres (Fra., Aquitaine)		N‐1		
SN1 /O	Estella‐Lizarra (Spa., Navarra)	H‐3	N‐2a	M‐2c	S‐5	FA16	Vieux‐Boucau‐les‐Bains (Fra., Aquitaine)	H‐1			
SN2	Murieta (Spa., Navarra)	H‐3				FA17	Pont‐de‐Lanne (Fra., Aquitaine)	H‐1			
SN3	Tafalla (Spa., Navarra)	H‐3	N‐2a	M‐2c		FA18 /C	Pissos (Fra., Aquitaine)	H‐1	N‐1	M‐1	S‐1
SN4	San Vincente (Spa., Navarra)	H‐3				FA19	Haut‐Richet (Fra., Aquitaine)	H‐1			
SN5	Sarries (Spa., Navarra)	H‐3				FA20	Bouglon (Fra., Aquitaine)		N‐1	M‐1	
SY1	Cerbon (Spa., Castilla Y Leon)	H‐3				FA21	Caudecoste (Fra., Aquitaine)	H‐1	N‐1	M‐1	
SY2	Fuentesauco (Spa., Castilla Y Leon)	H‐1				FA22	Campet (Fra., Aquitaine)	H‐1			
SY3	Arevalo (Spa., Castilla Y Leon)	H‐1		M‐1		FA23	St Chaliés (Fra., Aquitaine)	H‐1			
SY4	Veganzones (Spa., Castilla Y Leon)	H‐1		M‐1		FA24	Monpazier (Fra., Aquitaine)	H‐1			
SY5	Campaspero (Spa., Castilla Y Leon)	H‐1		M‐1		FA25	La Roche Chalais (Fra., Aquitaine)			M‐1	
SY6	Cuellar (Spa., Castilla Y Leon)	H‐1				FA26	Belin‐Beliet (Fra., Aquitaine)	H‐1			
SY7	La Pedreja (Spa., Castilla Y Leon)	H‐1	N‐1			FA27	Giscos (Fra., Aquitaine)			M‐1	
SY8	Valladolid (Spa., Castilla Y Leon)	H‐1				FA28 /B	Ychoux (Fra., Aquitaine)		N‐1	M‐1	S‐1
SY9	Valladolid (Spa., Castilla Y Leon)	H‐1				FM1	Nogaro (Fra., Midi‐Pyrénées)	H‐1	N‐1		
SY10	Tiedra (Spa., Castilla Y Leon)	H‐1				FM2	Verdun‐sur‐Garonne (Fra., Midi‐Pyrénées)	H‐3			
SY11	CervatosdiaCueza (Spa., Castilla Y Leon)		N‐1	M‐1		FM3	Monbarla (Fra., Midi‐Pyrénées)	H‐1			
SY12	Sotresgudo (Spa., Castilla Y Leon)	H‐1				FL1	Perpignan (Fra., Languedoc‐Roussillon)	H‐3	N‐2a	M‐2c	
SY13 /G	Cantalejo (Spa., Castilla Y Leon)		N‐1	M‐1	S‐2	FL2	Béziers (Fra., Languedoc‐Roussillon)		N‐2a		
SB1	Palma (Spa., Baleares)	H‐3				FL3 /H	Narbonne (Fra., Languedoc‐Roussillon)		N‐2a	M‐2c	S‐4
PN1	Olmos (Por., Norte)		N‐1			FL4 /I	Banyuls (Fra., Languedoc‐Roussillon)		N‐2a	M‐2c	S‐4
PN2a	Sanguinhedo (Por., Norte)	H‐1	N‐1	M‐1		FP1	Coubre (Fra., Poitou‐Charentes)	H‐1	N‐1		
PN2b	Sanguinhedo (Por., Norte)			M‐1		FP2	Châtellerault (Fra., Poitou‐Charentes)	H‐1	N‐1	M‐1	
PN3	Goios (Por., Norte)		N‐1	M‐1		FP3 /A	La Tremblade (Fra., Poitou‐Charentes)		N‐1	M‐1	S‐1
PN4	Moledo (Por., Norte)	H‐1		M‐1		FC1	Gemenos (Fra., Provence‐Alpes‐Côtes d'Azur)	H‐3			
SG1	Mos (Spa., Galicia)		N‐1			FC2	Marseille (Fra., Provence‐Alpes‐Côtes d'Azur)	H‐3		M‐2c	
SG2	Redondela (Spa., Galicia)	H‐1				FC3	Cassis (Fra., Provence‐Alpes‐Côtes d'Azur)	H‐3	N‐2a		
SG3	Soar (Spa., Galicia)	H‐1				MM1	Ifrane (Mor., Meknes)		N‐1	M‐3	
SG4	Oleiros (Spa., Galicia)	H‐1	N‐1			MM2	Ajabo (Mor., Meknes)		N‐1	M‐3	
SG5	Aldeavella (Spa., Galicia)	H‐1		M‐1		MT1	Asilah (Mor., Tanger)		N‐1	M‐3	
SG6	Aldeavella (Spa., Galicia)			M‐1		Out	*R. flavipes* ‐ Coubre (Fra., Poitou‐Charentes)		Outgroup		
SG7	Lires (Spa., Galicia)			M‐1							

At 15 of the 146 sites (coded with an additional letter – from A to O), transects were established to perform more extensive sampling. Along each transect, collection points were located at least 15 meters apart to maximize the number of different colonies sampled. Sites A, B, and C in southwestern France and sites G, D, and F in Spain have been previously studied by DeHeer et al. (2005) and Vargo et al. (2013), respectively; the other sites are reported for the first time in this study. A total of 341 individuals from these 15 sites were used to analyze the genetic structure of *Reticulitermes* populations.

### Chemical and statistical analyses (cuticular hydrocarbons)

To assess the geographical distribution of *R. grassei* and *R. banyulensis* chemotypes, cuticular hydrocarbon profiles were characterized using gas chromatography (GC), which separated out the different cuticular compounds (Table 1, code H‐x). The extraction, separation, characterization, quantification, and labeling of cuticular hydrocarbons were performed according to the methods described in Kutnik et al. (2004).

Multivariate principal component analyses (PCAs) using the relative proportions of the cuticular hydrocarbons were employed to differentiate clusters of chemical phenotypes within the species complex, as well as within each lineage. From the 98 interpretable chromatograms, we analyzed the 35 peaks shared by both species (Clément et al. 2001). The relative proportions of each peak were determined using the program Diamir (JMBS, Perkin Elmer Fr, Villebon‐sur‐Yvette, France); these data were then transferred into a Statgraphics matrix (Statgraphics v. 4.0 and Uniwin Plus v. 3.0, Francestat, France (www.statgraphics.fr)) to perform PCA. Of the 98 chromatograms analyzed, 61 were new to this study; 37 were published previously by Kutnik et al. (2004).

### DNA extraction, amplification, sequencing, and genotyping

For sequencing nuclear and mitochondrial genes, total genomic DNA was extracted from 86 individual termites as described in Kutnik et al. (2004). Different programs and amplification conditions were employed depending on the types of primers used for sequencing (Table S1). Most of the successfully amplified samples were originally stored only in alcohol or were removed from pentane after cuticular lipid extraction.

Three genes commonly used to study the phylogeny of the *Reticulitermes* genus were investigated: the mitochondrial cytochrome oxidase I gene (COI) and the mitochondrial cytochrome oxidase II gene (COII; 65 individuals; Table 1; code M‐x), as well as the nuclear ITS2 region (internal transcribed spacer) of the ribosomal DNA (59 individuals; Table 1; code N‐x). All of the COI sequences analyzed were new to this study as were 39 of the 65 COII sequences (the other 26 were from Kutnik et al. 2004). Of the 59 ITS2 sequences analyzed, 38 were new to this study; the other 21 were reported in Kutnik et al. (2004).

Amplified DNA was purified using the Qiagen PCR Purification Kit and cycle‐sequenced in both directions using an automated AB *3100‐Avant* sequencer. Each sequence generated by this study was deposited in the GenBank database (accession numbers: JQ431045 to JQ431094 and KP721513 to KP721527 for COI sequences; JQ430995 to JQ431043 and KP721528 to KP721542 for COII sequences; and JQ431095 to JQ431133 and KP721543 to KP721557 for ITS2 sequences [see Table S2]).

To characterize population genetic structure, we used ten microsatellite loci: *Rf6‐1*,* Rf15‐2*,* Rf21‐1*,* Rf24‐2*,* RS1*,* RS10*,* RS15*,* RS62*,* RS76*, and *RS78* (Table 1; code S‐x). Microsatellite loci had previously been isolated from *R. flavipes* (Vargo 2000) and *R. santonensis* (Dronnet et al. 2004; DeHeer et al. 2005); the latter is a French population of *R. flavipes* recently synonymized with *R. flavipes* (Austin et al. 2005; Perdereau et al. 2010, 2013). The amplification of all primers was performed using a Stratagene thermal cycler in accordance with Vargo (2000), except for the following minor modifications. Amplification of *Rf15‐2*,* RS62*, and *RS76* was performed in a single reaction where the final primer concentrations were 50 *μ*mol/L for *Rf15‐2*, 70 *μ*mol/L for *RS62*, and 30 *μ*mol/L for *RS76*. The annealing temperature was increased to 57°C for *RS76*,* RS62*, and *Rf15‐2* to eliminate nonsense bands that interfered with scoring. *Rf24‐2* and *Rf6‐1* were amplified in multiplexed reactions, with primer concentrations of 30 *μ*mol/L for *Rf24‐2* and 100 *μ*mol/L for *Rf6‐1*. PCR products were separated by electrophoresis on 6% polyacrylamide gels run on a Li‐Cor 4000L DNA sequencer; allele sizes were determined by comparison to a 50–350 bp IRDye^™^ 800 standard (LiCor, Inc., Lincoln, NE). Alleles were scored using the computer program gene profiler 4.03 (Scanalytics, Inc., Milwokee, WI).

### Analyses of mitochondrial data

The mitochondrial sequences (COI and COII) were concatenated to form single sequences that were then aligned using the ClustalW algorithm (Thompson et al. 1994) in the BioEdit v. 4.8.10 sequence editor (Hall 1999). Phylogenetic analyses were then performed using three methods: a phenetic approach using neighbor‐joining (NJ) (Saitou and Nei 1987), a cladistic approach using maximum parsimony (MP) (Tamura et al. 2011), and a probabilistic approach using Bayesian inference (BI). The NJ tree was constructed using a Kimura 2‐parameter model of substitution following the methods of Austin et al. (2004). The MP tree was constructed using the close‐neighbor‐interchange branch swapping algorithm and 100 repetitions of random addition sequences in MEGA v. 4.0.2 (Tamura et al. 2011). Branch support was evaluated by performing 1000 nonparametric bootstrap replicates for the three methods, and all sequences were analyzed using an *R. flavipes* consensus sequence as outgroup. Finally, BI analyses were conducting using a partition‐by‐gene (COI and COII genes) scheme for combining the mitochondrial alignments. The most likely models for each gene were determined using MrModeltest v. 2.0 (Nylander 2004) and employing the Akaike Information Criterion; the best‐fit models selected were GTR+I and HKY+I for COI and COII, respectively. A Bayesian search was carried out using MrBayes v. 3.1.2 (Huelsenbeck and Ronquist 2001) and employing four simultaneous Markov chains, 10,000,000 generations, and a sampling frequency of 100 generations, which resulted in 100,000 generations being saved. A burn‐in setting of 20% was used. We used Tracer v. 1.5 (Drummond et al. 2012) to confirm that our effective sample size was adequate for estimating the posterior distribution.

To complement the traditional phylogenetic approaches used, a haplotype network was also constructed using TCS v. 1.21 (Clement et al. 2000) to characterize the genetic relationships among colonies and estimate the mutational distances between the lineages. Parsimony calculations were conducted using the 95% connection limit default setting.

### Analyses of nuclear genetic data

The level of intraspecific polymorphism in ITS2 genes is low in European *Reticulitermes* termites and is often used to quickly identify different taxa (Jenkins et al. 2001; Uva et al. 2004). We therefore used it here as a quick and reliable marker with which to flesh out the taxonomic identity and compared the results it yielded with those of the other markers used.

Termite populations on the Iberian Peninsula and in southern France were analyzed using 10 microsatellite loci for the 15 more intensively sampled sites (A to O). We studied 16–33 colonies per site using one individual per colony. The program Genetix 4.05 (Belkhir et al. 1996) was used to calculate expected heterozygosity and the mean allele number per locus for each population. To infer population structure, the multilocus genotypes obtained were analyzed using a model‐based clustering method implemented in the program STRUCTURE (Pritchard et al. 2000) with no a priori assumption of population structure. Under this method, individuals are probabilistically assigned to each cluster based on the proportion of their genome that matches that cluster. STRUCTURE analysis was performed assuming the admixture model with allele frequencies correlated. Runs were based on 50,000 iterations after a 50,000 burn‐in period of the Markov chain with *K* set from 2 to 15, replicated 20 times to check concordance of the data. The optimal value of *K* was calculated using log likelihood (Pritchard et al. 2000) and delta *K* (Evanno et al. 2005). Because populations could potentially hybridize, the admixture model (Pritchard et al. 2000) was used.

To determine whether there was evidence of a genetic bottleneck (i.e., a reduction in genetic diversity caused by a recent decrease in effective population size), the multilocus genotypes described above for each of the intensively sampled 15 populations were evaluated for heterozygote excess using the program BOTTLENECK v. 1.2.02 (Piry et al. 1999). Three tests (a sign test, an SD test, and a Wilcoxon sign‐rank test) were performed under two mutation models: an infinite allele model (IAM) and a two‐phased model (TPM) in which 70% of the mutations followed a single‐step mutation model and 30% produced multistep changes.

## Results

### Chemical polymorphism

**Figure 2:**
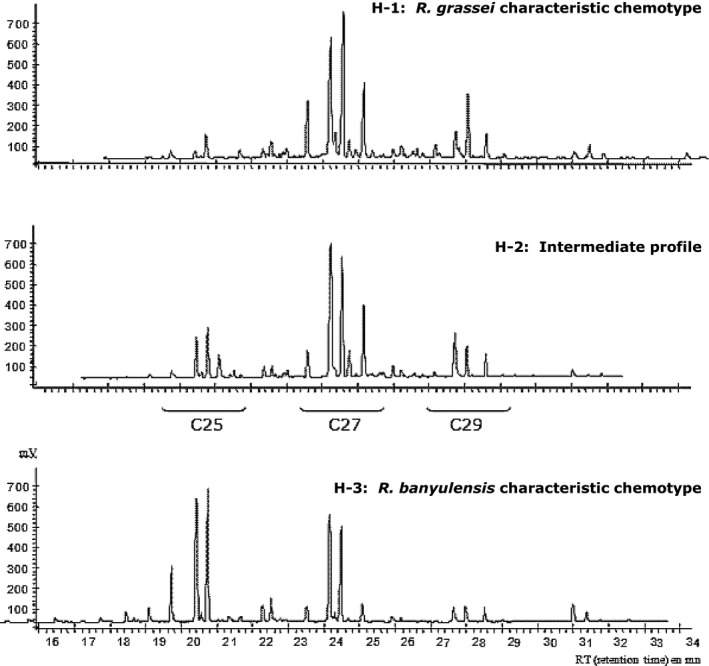
Representative chemical profiles of the different taxa. Characteristic cuticular hydrocarbon chemotypes for *R. grassei* and *R. banyulensis*, as well as an intermediate profile from a Basque Country colony that showed groups of peaks (C25, C26, C27) common to each of the two lineages.

**Figure 3:**
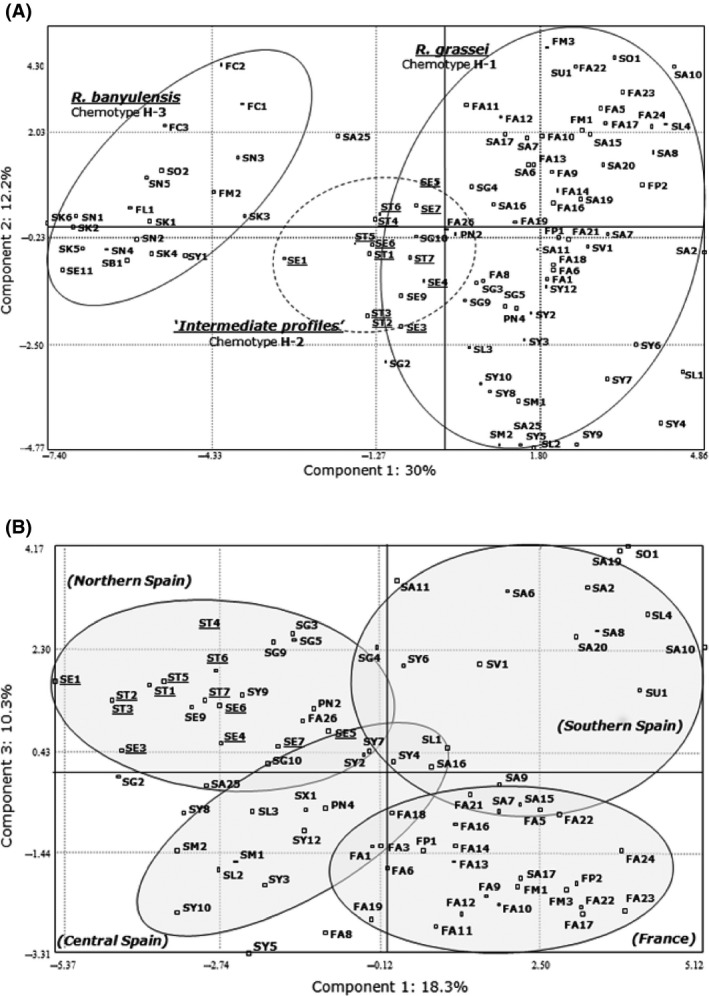
(A) Principal component analysis performed using 98 colonies from the two lineages. Colonies with *R. grassei*‐like or *R. banyulensis*‐like chemotypes are encircled by solid lines. The colonies with intermediate profiles are underlined and encircled by dotted lines. (B) Principal component analysis performed with only the *R. grassei* samples. The ellipses distinguish the different geographical clusters of *R. grassei* on the Iberian Peninsula and in southwestern France. The colonies with intermediate profiles are underlined.

Cuticular hydrocarbons form a chemical signature that is commonly used as a phenotypic marker to differentiate termite species, castes, or colonies. Using the 98 collected chromatograms, profiles characteristic of *R. grassei* versus *R. banyulensis* (chemotypes H‐1 and H‐3, respectively) were identified. However, we also obtained a profile that, visually, seemed to be intermediate (chemotype H‐2; Fig. 2). Multivariate analyses (principal component analyses [PCAs]) using all the colonies were then performed; they confirmed the distinction between the three chemical profiles. In the first analysis (Fig. 3A), the first two canonical axes accounted for 42.2% of the variation and revealed two different groups corresponding to the two termite lineages. The *R. grassei* and *R. banyulensis* profiles (H‐1 and H‐3) were indeed well separated and found on both sides of the first axis (30% of the variation), while the chemical profile (H‐2) of individuals from northern Spain were located between them. This intermediate profile previously reported by Kutnik et al. (2004) appears even more distinct due to the greater number of samples analyzed (98 compare to 37). Moreover, when we performed a PCA using only *R. grassei* samples (H‐1 and H‐2), we were able to distinguish four different geographical clusters (Fig. 3B). The colonies from southern Spain seem to be more dispersed than those of northern Spain (including the intermediate individuals), central Spain, or France.

### Mitochondrial sequences

**Figure 4:**
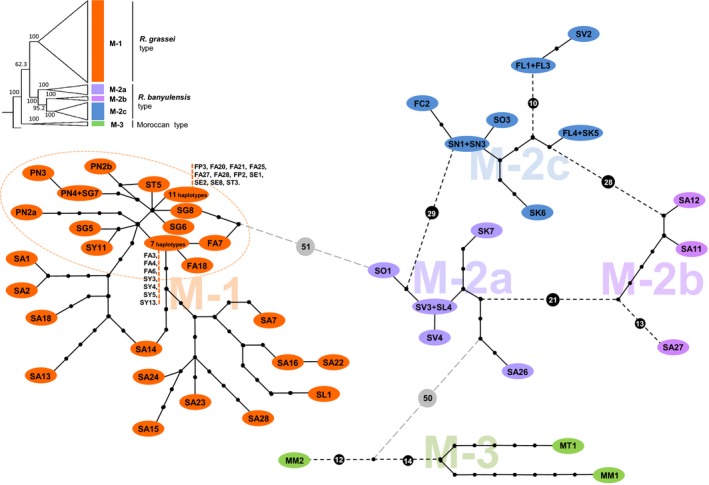
Minimum‐spanning networks of mtDNA haplotypes (COI–COII concatenated genes) combined with a Bayesian phylogenetic analysis. Each haplotype is represented as an oval whose color corresponds to the phylogenetic clade to which it was assigned via Bayesian analysis (cf. Fig. S1). Each solid line segment between the haplotypes represents a single mutational step, and each solid dot represents the inferred intermediate haplotype. However, for clarity, segments between solid dots were not included and the genealogical links between haplotypes that exceeded 10 mutations are represented by dashed segments on which the number of inferred intermediate haplotypes is indicated. The dashed circle is used to group samples from northern Spain belonging to the M‐1 subclade.

The concatenation of the COI and COII mitochondrial sequences resulted in the construction of 1492‐bp sequences from 65 colonies. Regardless of the method used, colonies are clearly partitioned into two major clades, M‐1 and M‐2 (corresponding to what we called clade 1 and 2 in (Kutnik et al. 2004); Figs. 4, S1). These analyses also produced some new insights. Indeed, a third clade, M‐3, was formed by the Moroccan populations. Genetic partitioning is strong for the three groups; node support approached 100%. The Moroccan clade occupies a basal position according to all the analytical methods we used (NJ, MP and BI). The M‐1 clade (*R*. *grassei*), which contains individuals from 43 locations, cannot be split into different subclades. In contrast, the M‐2 clade (*R*. *banyulensis*), which contains individuals from 19 locations, can be split into three well‐supported subclades, called M‐2a, M‐2b, and M‐2c (Table 1; Figs. 4, S1).

At the nucleotide level, the *R. banyulensis* clade (M‐2) was the most variable; there were 47 informative sites for the 66 variable positions. The *R. grassei* clade (M‐1) had 26 informative sites for 49 variable positions. The differentiation between the two clades was also clearly established by 34 fixed substitutions. The Moroccan clade had 20 unique substitutions. Of the 34 substitutions distinguishing the two‐first clades, 12 are shared by the Moroccan and *R. grassei* clades and 22 are shared by the Moroccan and *R. banyulensis* clades.

The mutational distances among the clades were further revealed by the haplotype network (Fig. 4). Three haplotype groups (M‐1, M‐2 and M‐3) are clearly present in the network; they correspond to the three clades observed in the phylogenetic analyses. The genealogical relationships among the haplotypes were reconstructed using a 90% cutoff limit for the connection between haplotypes. Most are singletons, with the exception of seven (Fig. 4). The divergence among the three groups reveals that mutation levels are comparable: The M‐1 and M‐3 groups diverge from the M‐2 group by 51 and 50 bp, respectively. Within *R. grassei* (M‐1 clade), the haplotypes form a condensed network, particularly those from northern and central Spain (encircled by a dotted line in Fig. 4). In contrast, the *R. banyulensis* haplotypes (M‐2 clade) are more scattered and cluster into three phylogenetic subclades (Fig. 4). The M2‐a colonies (clear blue) from southeastern Spain (province of Valencia) are particularly interesting as they occupy a central position in the haplotype network and are connected to other clades.

**Figure 5:**
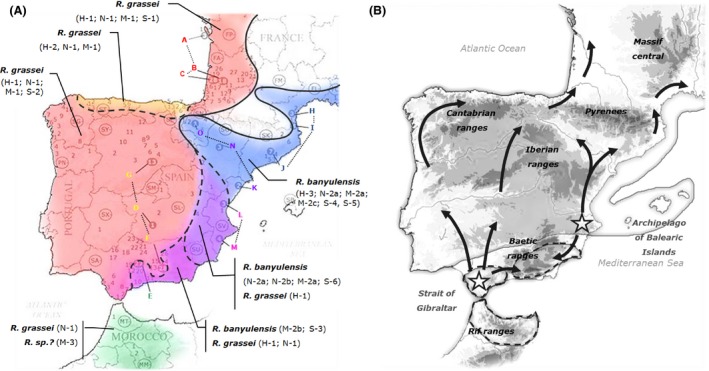
(A) Map showing the present biogeography of the Iberian *Reticulitermes* species complex. The areas where the different genetic assemblages were detected are delimited and labeled. The sampling sites (letters from A to O) that are linked by dotted lines depict the populations revealed by the genetic structure analysis. (B) Map illustrating the hypotheses regarding the origins and postglacial expansion of the two Iberian *Reticulitermes* lineages. The stars indicate the two possible glacial refugia associated with each lineage. The arrows indicate the possible postglacial colonization routes. The areas encircled by dotted lines represent the paleogeological islands where Iberian *Reticulitermes* lineages may have started to diverge following a vicariance event. The coastline delimitation of these islands and of the continent during the Tortonian (~12 Myr ago) are traced according to the topography proposed by De Jong (1998). The names of the sea and the mountain ranges are also indicated on the map.

More generally, both the phylogenetic and network analyses provide important insights into Iberian *Reticulitermes* biogeography. The M‐2 haplotypes are distributed across the entire Mediterranean coast, from southern Spain to southern France, and along the Ebro valley in northeastern Spain. In contrast, the M‐1 haplotypes cover the rest of the Iberian Peninsula, from the southern edge of Spain to southwestern France (Fig. 5A).

### Nuclear genes

#### ITS2 Region

**Figure 6:**
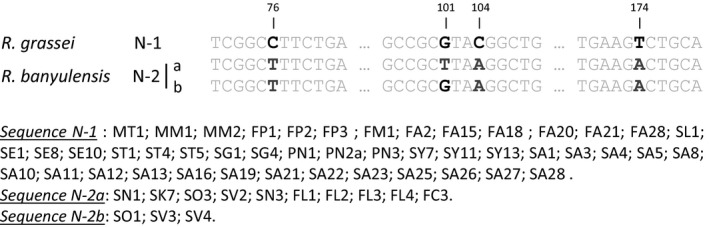
Polymorphic ITS2 nuclear sequences with the variable nucleotide positions highlighted in black and gray.

As expected and already noted by Kutnik et al. (2004), the two Iberian lineages exhibit very little polymorphism in the sequenced ITS2 region; they differ at only four nucleotide positions (1%) out of a total of 372 bp. Of the 59 genotypes sequenced, we found 46 N‐1, 10 N‐2a, and 3 N‐2b, which generally matched the chemotype results. Three exceptions were found: colonies SO1, SV3, and SV4 (N‐2b), from southeastern Spain (i.e., province of Valencia), differed at only three positions from the characteristic *R. grassei* sequence (N‐1; Fig. 6). In contrast to their chemical signatures, these colonies more closely resemble *R. banyulensis* genetically, sharing the characteristic sequence (N‐2a) found in 10 other colonies from southeastern France and northeastern Spain (Fig. 6). The remaining 46 colonies shared the sequence associated with *R. grassei* (N‐1; Fig. 6). These samples were widely distributed across three‐quarters of the Iberian Peninsula, from southern Spain to northwestern Spain and southwestern France (Fig. 5A). Unexpectedly, this group also included the three samples from northern Morocco (MT1, MM1, and MM2). All the samples exhibiting the N‐1 phylotype fit the *R. grassei* chemotype H‐1. Only the SO1 sample from Valencia province had the H‐1 chemotype (*R. grassei*) with the N2b phylotype rather associated to *R. banyulensis* (Fig. 6).

#### Microsatellite genotypes

**Figure 7:**
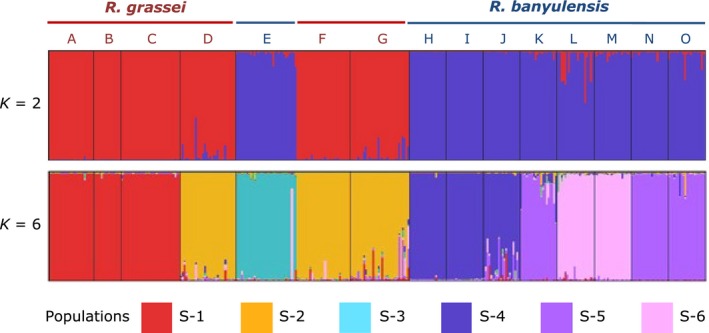
Inferred population structure of southwestern European *Reticulitermes* termites based on the analysis of 10 microsatellite loci using samples collected at 15 sites (A‐O). Each individual (representing a site) is depicted using a thin vertical line and probabilistically assigned to *K* populations (colored segments). Bar plots were generated for K = 2 to K = 7 (cf. Fig. S2), but only two runs are shown here. The first bar plot shows the clear distinction between the two lineages – *R. grassei* and *R. banyulensis*; the second shows the assignment of *K* = 6 genetic populations as identified by the STRUCTURE analysis.

Extensive sampling was also conducted in parallel within smaller areas throughout the distribution ranges of *R. grassei* and *R. banyulensis*. The goal was to obtain a data set more suitable to the analysis of population genetic structure (Ross et al. 2007; Caldera et al. 2008). A total of 341 termites from 15 sites (A to O; Table 1 and Fig. 1) were examined. A total of 121 alleles were detected across the 10 microsatellite loci. The main results of the STRUCTURE analysis (*K* = 2 and *K* = 6) are shown in Figure 7, but the histograms for the relevant runs from *K* = 2 to *K* = 7 are provided in Figure S2. The most likely number of populations was estimated at *K* = 6 using the calculation of different criteria as per Evanno et al. (2005) (Fig. 7). However, these graphical indicators did not yield clear results, and biological and ecological interpretation is required to delineate population genetic structure (Evanno et al. 2005). For *K* = 2, STRUCTURE revealed the existence of two groups that corresponded to the two lineages, *R. grassei* (A to D, F, and G) and *R. banyulensis* (E and H to O). For *K* = 3, *K* = 6, and *K* = 7, *R. banyulensis* colonies were split into several populations along the Mediterranean coast (Fig. S2). In contrast, and despite the geographical distance, *R. grassei* colonies formed a single population until *K* = 5, at which point the French (A, B, C) and Spanish (D, F, G) sampling sites clustered into two distinct populations (Fig. S2). It is worth mentioning that neither mixed‐species colonies nor hybrid individuals were found (DeHeer et al. 2005; Vargo et al. 2013).

**Figure 8:**
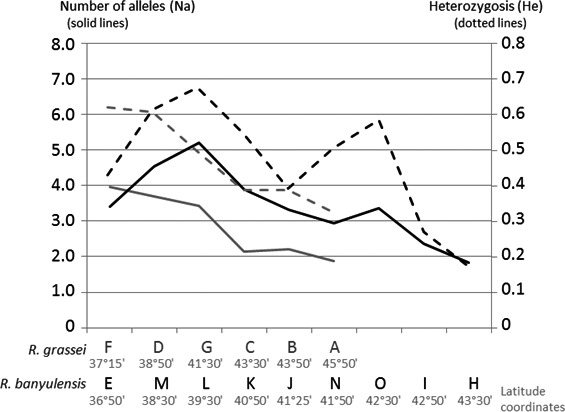
Changes in genetic diversity along a south–north gradient. Allele number (solid lines) and heterozygosity (He, dotted lines) are shown; populations of *R. grassei* are in gray, and populations of *R. banyulensis* are in black.

A more detailed examination of the genetic parameters yields further insights into population structure and biogeographical history. For all the samples and loci, mean heterozygosity (He) was equal to 0.47, with an average allele number of 3.21. However, allele number and heterozygosity declined along a south–north gradient (Fig. 8). At the *R. grassei* sampling sites (gray curves, Fig. 8) in southern Spain (F) and southwestern France (A), number of alleles varied from 4.0 to 1.83, respectively. Mean heterozygosity varied from 0.62 to 0.33, respectively. For *R. banyulensis* colonies (black curves, Fig. 8), the most genetically polymorphic site was not located at the extreme edge of southern Spain, but rather in the east, near Valencia (site L: He = 0.68, allele number = 5.20). (F) and southwestern France (A), mean allelic richness varied from 5.20 to 1.83, respectively. Mean heterozygosity varied from 0.62 to 0.33, respectively. For *R. banyulensis* colonies, the most genetically polymorphic site was not located at the extreme edge of southern Spain, but rather in the east, near Valencia (site L: He = 0.68, allele number = 5.20; blue curve, Fig. 8). Genetic diversity was lost toward the south (E: He = 0.43, allele number = 3.40) and the north, in the direction of the French populations (H: He = 0.17; allele number = 1.83). To a great extent, all the French populations of both lineages exhibited low polymorphism and may have undergone a genetic bottleneck in the recent past, perhaps when the Pyrenees were crossed. This hypothesis was tested using BOTTLENECK software (Piry et al. 1999). The three statistical tests (sign test, SD test, and Wilcoxon sign‐rank test) found a significant level of heterozygote excess (*P* < 0.05) under the two mutation models (IAM and TPM), as expected for the French sites. As for the Spanish sites, only site D had significant *P*‐values for the three tests.

## Discussion

### Time of divergence and origins of the two lineages

#### Time of divergence

A recent Bayesian phylogenetic analysis of the genus *Reticulitermes* worldwide (Dedeine et al. 2016) estimated the divergence time between *R. grassei* and *R. banyulensis* at ca. 7 Mya. This is much earlier than the estimate of Kutnik et al. (2004) who placed the genetic differentiation of these two lineages in the middle of the Pleistocene (i.e., before the last ice age, Würm, 20,000 years ago). This estimated time of divergence of the Iberian termite lineages during the Miocene appears to be consistent with a possible vicariance event. Indeed, the paleogeographical reconstruction of Iberian Peninsula shorelines in the Tortonian has revealed the existence of three “fossil” islands spread across the current mountain ranges of the Sierra Nevada and the Province of Cadiz in Spain and the Rif region in Morocco (Fig. 5B; De Jong 1998; Jiménez‐Moreno et al. 2010; Jolivet et al. 2006). These fossil islands, which existed until the tectonic closure of the Strait of Gibraltar during the Messinian salinity crisis (around 5.5 Myr; De Jong 1998), could have resulted in the isolation of the ancestral populations of both *R. banyulensis* and *R. grassei* over a prolonged period. This hypothetical situation is consistent with an allopatric speciation model. One of the “fossil” islands corresponds to the current Moroccan Rif mountain range. At this stage, it is difficult to determine the specific nature (i.e., ancestral or not) of the Moroccan populations and clarify their phylogeographic relationships with the European *Reticulitermes* termites. The *R. grassei*‐like nuclear genome of the Moroccan colonies might be the result of recent introductions, incomplete lineage sorting, or convergent evolution of the ITS regions. In any event, these findings indicate that intimate relationships exist among termites found on both sides of the Strait of Gibraltar as in other insect species (Horn et al. 2006; Rodrigues et al. 2014). They also suggest that the key to understanding the origin of the *R. grassei*/*R. banyulensis* complex may lie in North African populations. The hypothesis of an African origin for Iberian *Reticulitermes* is indeed intriguing but needs to be tested using more extensive research on the chemical and genetic compositions of North African populations.

#### Glacial refugia in the Pleistocene

As much as the paleogeography of the late Tertiary has been helpful in building a coherent scenario for speciation in *Reticulitermes*, it is likely that the Quaternary glacial cycles better explain the present‐day genetic structure of Iberian populations. Indeed, the high rates of polymorphism and heterozygosity observed for microsatellite markers in populations in southern Spain (Fig. 8) could indicate the use of ice age refugia rather than the presence of species cradles, that is, geographical sources of origins of the species. The high genetic diversity found in the Andalusian population (E) could indicate that the southern tip of Spain might be the place where ancestral populations of *R. grassei* persisted during the last glacial maximum. Similarly, for the *R. banyulensis* clade, the highest level of polymorphism is found northeast of the Baetic mountain ranges, in the province of Valencia (L; Fig. 8). Furthermore, colonies from this region are found in the center of the mitochondrial haplotype network (Fig. 4). It is therefore possible that this region served as a major ice age refugium for this lineage (Fig. 5B), as it did for other plant and animal lineages (Gómez and Lunt 2007; Médail and Diadema 2009; Campillo et al. 2011; Miraldo et al. 2011; Abellán and Svenning 2014). This refugium is further to the north and east that what proposed by Kutnik et al. (2004) suggesting the Andalusia refugium was a separate southern refugium. The genetic relationships among termites from the nearby Balearic Archipelago merits investigation as gene flow between these islands and the Iberian Peninsula could explain the high level of genetic diversity detected at site L. Moreover, the Balearic Islands could also have been glacial refugia (López de Heredia et al. 2007; Planas et al. 2014).

### The different routes of postglacial colonization

#### Colonization of the Iberian Peninsula

The glacial cycles of the Pleistocene were marked by several warming periods that eventually led termite populations to emerge from their southern refugia and spread throughout the entire Iberian Peninsula until they established their present‐day distribution. Based on our results, it is probable that these populations followed complicated routes of postglacial colonization that were constrained by topography, climate, and vegetation (Crowley and North 1991; Fig. 5B). The *R. grassei* lineage may have dispersed from southern Spain along two migration fronts, one that followed the Atlantic coast and the other that traced a more or less “straight line” north, crossing the center of the peninsula. This hypothesis would explain the simultaneous presence in France of two populations of termites that, on the one hand, exhibit the mitotypes found in the center of the peninsula, and on the other hand, display mitotypes related to those occurring on the northern coast of Spain (Figs. 4, S1). Furthermore, the *R. banyulensis* lineage likely spread along the Mediterranean coast to both the south and the north, as well as along the Ebro Valley. These migration events would most likely have been slow for subterranean termites because of their limited dispersal abilities and because of the presence of numerous natural obstacles (mountains, rivers, vegetation), resulting in a gradual decline in genetic diversity (Hewitt 1996). We observed a decrease in both the number of microsatellite alleles and the levels of heterozygosity (Fig. 8), as well as a decline in the variability of the mitochondrial sequences (Fig. 4). This pattern is consistent with a previously proposed migration model for temperate European insects: they are hypothesized to have dispersed from southern ice age refugia (Hewitt 1996). It also matches the eastern/western postglacial colonization routes followed by many tree species on the Iberian Peninsula (Rodríguez‐Sánchez et al. 2010), particularly the pine species that *Reticulitermes* termites consume.

#### Crossing of the Pyrenees and recent dispersal

The Pyrenees's mountains at the frontier of Spain and France constitute a very important dispersal barrier that is reflected in the genetic structure of termite populations, as well as in that of many other species (Gómez and Lunt 2007; Miguel et al. 2007; Milá et al. 2013). When termites traversed this mountain range, they appear to have experienced a significant loss of genetic diversity due to a bottleneck effect. As a result, the French populations of both lineages exhibit strong genetic and chemical homogeneity despite their broad geographical ranges. *R. grassei* has spread naturally along the Atlantic coast as far north as the La Coubre forest (Department of Charente‐Maritime), a distance of over 400 km (Bankhead‐Dronnet et al. 2015). *Reticulitermes banyulensis* has reached the coast of the departement of Alpes‐Maritimes (close to Nice) and might even extend up the Rhône valley to the Gard (department of Gard), over a distance exceeding 300 km (Bagnères & Dupont, unpubl. data). In addition to their dispersal by natural means, the two lineages have also had their ranges artificially extended by human‐mediated dispersal. For example, *R. grassei* colonies have been found in Devon, England (Jenkins et al. 2001) and northern France (Bagnères & Dupont, unpubl. data), and *R. banyulensis* occurs in Lyon (department of Rhône; Bagnères & Dupont, unpubl. data), the third largest city in France, approximately 200 km north of the Mediterranean habitat where it is naturally found. Introduction events in the natural environment can be difficult to detect, especially if they occurred long ago and close together (Nobre et al. 2006).

#### Presence of mixed patterns in contact zones

Since their isolation in southern Spain, *R. grassei* and *R. banyulensis* have evolved separately via opposing colonization pathways. However, the existence of possible areas of sympatry in Andalusia, eastern Castile, and at the Navarra/Basque Country border raises questions about hybridization. Clément (1982) excluded this possibility after conducting behavioral experiments on French termites that showed that reproductive alates from each lineage preferentially paired with conspecifics. This reproductive isolation is strengthened by a high level of intertaxon aggression between workers, which prevents breeding between reproductives (Bagnères et al. 1991). However, in the present study, we detected three distinct geographical areas on the Iberian Peninsula where marker patterns were mixed (Fig. 5A). First, from Asturias to the Basque Country, termites from 20 colonies had *R. grassei* genotypes but intermediate chemical phenotypes (H‐3, Table 1) – they had some major cuticular hydrocarbons from each lineage (Fig. 2). Second, in Andalusia, there was a sympatric area in which *R. grassei* colonies and mixed colonies co‐occurred. The latter resembled *R. grassei* in their chemical signatures and ITS2 markers, and resembled *R. banyulensis* in their mitochondrial (Salares SA11, Antequera SA12, Belmez SA26, and Lucena SA27 colonies) and microsatellite markers (Lucena SA27 colony; Table 1). Finally, along the Mediterranean coast, surrounded on either side by the Iberian and Baetic ranges, some termite colonies had all the markers of the *R. banyulensis* clade, with the exception of their cuticular hydrocarbon signatures, which were like those of *R. grassei* (Casas Ibanes SL4 and Mas de Jacinto SO1 colonies; Table 1).

The presence of chemically intermediate colonies between Asturias and the Basque Country suggests that genes might be exchanged between the two lineages. However, it is difficult to conclude that introgression is occurring solely based on chemical data because the relative roles of genetic and environmental factors in shaping insect cuticular signatures remain poorly understood. Diet has been implicated in several species (Matsuura 2001; Rouault et al. 2004; Buczkowski et al. 2005). Similarly, other research has shown that a termite colony's signature can change rapidly if contact with another species occurs (tests conducted on mixed colonies of *R. grassei* and *R. [santonensis] flavipes* (Bagnères et al. 1991; Vauchot et al. 1996). The biosynthetic pathway that results in the cuticular signature may be regulated by genes that evolve rapidly, as seen in crickets (Mullen et al. 2007) and in different populations and species of *Drosophila* influenced by different biotic and abiotic factors (Frentiu and Chenoweth 2010; Bontonou et al. 2013). However, our results support the hypothesis that gene flow is occurring between the two lineages, and several factors may explain the unique and perhaps hybrid intermediate chemical profile. First, the geographical proximity of *R. banyulensis* and *R. grassei* colonies in Navarra means some interspecific contact is possible, even if it may be limited by intertaxon aggressiveness. Second, genetic diversity peaked at site O, which could be due to gene flow from nearby colonies of the sister lineage (Fig. 8).

The mismatched nuclear and mitochondrial genotypes observed in some Andalusian colonies suggest possible asymmetric introgression. This pattern is similar to that observed in the *R. lucifugus/R. l. corsicus* complex found in Tuscany, Italy (Lefebvre et al. 2008) and provides evidence for relatively ancient hybridization events. In this case, reproductive barriers between the two taxa may have been surmounted either by the rare occurrence of heterospecific alate pairing (Clément 1982) or by interbreeding between neotenics resulting from the fusion of heterospecific colonies due to reduced interspecific aggression. Indeed, the results of behavioral tests conducted on populations in southern Spain support this latter possibility (Kutnik 2004).

#### Population structure

Analysis of population genetic structure can provide an integrated picture of both the phylogeography of *R. grassei* and *R. banyulensis* and the contemporary processes of dispersal and gene flow that shape the two taxa (Vargo and Husseneder 2011). The genetic structure illustrated in Figures 7, S2 reveals a strong east–west separation that matches the clustering of the two lineages. Indeed, the region's mountain ranges serve to geographically isolate the French populations beyond the Pyrenees and the *R. banyulensis* populations along the Mediterranean coast. These populations are fragmented in the areas where the intermediate forms described above occur. The present‐day biogeography of the two lineages is thus a product of the region's topography and diverse climates, as well as of the populations' evolutionary histories *(e.g*., their different glacial refugia and colonization routes). The population structure of these two lineages has no doubt also been influenced by isolation and dispersal processes that occurred during the Pleistocene's multiple glaciation/warming cycles, as is the case for other species in the region (Paulo et al. 2002; Hewitt 2004; Gómez and Lunt 2007; Abellán and Svenning 2014; Gonçalves et al. 2015; Rato et al. 2016). Consequently, the distribution of termite populations described here is consistent with the geographical division (biotic sectors) observed for both animal and plant groups endemic to the Iberian Peninsula (García‐Barros et al. 2002).

## Conclusion

Our study used the analysis of four genetic and chemical markers to more fully describe the present biogeography of the two Iberian termite lineages – *R. grassei* and *R. banyulensis* – and to infer the historical processes responsible for their current distributions and evolutionary relationships. Considered separately, each marker reflects changes on different timescales, yielding a distinct and fragmented evolutionary history. However, taken together, the markers reveal a more complete picture. The strong genetic and phenotypic divergence between the two lineages have their origins in vicariance events associated with Pleistocene glacial refugia and perhaps with even earlier Miocene paleogeological islands. The two lineages are not completely reproductively isolated as interspecific breeding appears to have occurred in sympatric areas following postglacial expansion. However, in the French populations, we never observed intermediate forms or evidence of introgression. This result suggests that following the crossing of the Pyrenees, the speciation process separating *R. grassei* and *R. banyulensis* was completed. Thus, Iberian *Reticulitermes* speciation appears to have occurred in two steps. First, geographical isolation in southern Spain resulted in some allopatric differentiation, and second, the arrival in southern France at the end of the postglacial expansion resulted in parapatric speciation (Fig. 5B). Based on all available evidence to date, it now appears that the species status of these two lineages, which has long been debated, can be confirmed, at least in the case of the French populations. *R. grassei* and *R. banyulensis* are well defined by various taxonomic criteria (morphological, behavioral, chemical, and genetic; Clément and Bagnères 1998; Clément et al. 2001; Kutnik et al. 2004; Nobre et al. 2006, 2009; Leniaud et al. 2011). This study has found that their complex evolutionary histories (marked by some potential hybridization events) and their resulting distributions are also distinct. Of the two species, the colony breeding structure of *R. grassei* is now better understood (DeHeer et al. 2005; Nobre et al. 2008). We recently studied additional samples of the two species to expand our knowledge. In particular, we examined various populations in southwestern France (Bankhead‐Dronnet et al. 2015) and the genetic structure of colonies of both species that occur across the Iberian Peninsula (Vargo et al. 2013).

## Conflict of Interest

None declared.

## Data accessibility

GenBank accession numbers are in the supporting information part, and sampling locations are given in Figure 1 and Table 1 in the main manuscript.

## Supporting information


**Table S1.** Primers and PCR programs used for the different genetic analyses.
**Figure S1.** Bayesian phylogenetic tree inferred from the mitochondrial COI‐COII concatenated sequences.
**Figure S2.** Population structure of southwestern European *Reticulitermes* termites based on the analysis of 10 microsatellite loci at 15 locations (A‐O).
**Table S2.** Table with Genbank numbers for each site, the lab codes used in Kutnik et al. (2004), and the codes used in the present study.
