# Supplementary material for: Subterranean termite phylogeography reveals multiple postglacial colonization events in southwestern Europe
Source: Ecol Evol. 2016 Jul 27;6(16):5987–6004. doi: 10.1002/ece3.2333 (PMC4983608; doi:10.1002/ece3.2333)
Supplement: Supplementary file 1 — Table S1. Primers and PCR programs used for the different genetic analyses. Figure S1. Bayesian phylogenetic tree inferred from the mitochondrial COI‐COII concatenated sequences. Figure S2. Population structure of southwestern European Reticulitermes termites based on the analysis of 10 microsatellite loci at 15 locations (A‐O). Table S2. Table with Genbank numbers for each site, the lab codes used in Kutnik et al. (2004), and the codes used in the present study. [file ECE3-6-5987-s001.docx]

**Subterranean termite phylogeography reveals multiple postglacial colonization events in southwestern Europe**

**Thomas Lefebvre ^a,¥^, Edward L. Vargo ^b^, Marie Zimmermann ^a^, Simon Dupont ^a^, Magdalena Kutnik ^a,c^, Anne-Geneviève Bagnères ^a,^***

***Supporting Information***

**Table S1**: Primers and PCR programs used for the different genetic analyses.

**Figure S1**: Bayesian phylogenetic tree inferred from the mitochondrial COI-COII concatenated sequences. The numbers at the nodes indicate bootstrap values for the NJ, MP, and BY analyses, respectively. The absence of a bootstrap value (marked by a dash [-]) means that tree topology is different for that branch and method or the bootstrap value is below 50%. Clade labels are on the right: the two Iberian *Reticulitermes* lineages plus a Moroccan branch are present.

**Figure S2**: Population structure of southwestern European *Reticulitermes* termites based on the analysis of 10 microsatellite loci at 15 locations (A-O). In contrast to Figure 6, more STRUCTURE runs, from K=2 to K=7, are represented here.

**Table S2**: Table with Genbank numbers for each site, the lab codes used in Kutnik et al. (2014), and the codes used in the present study.

**Table S1**

|  | Locus | Primers | Sequence (5’ > 3’) | Source | PCR profiles |
| --- | --- | --- | --- | --- | --- |
| mitoch. | COI | COI F 2195 | TTG ATT CTT TGG TCA CCC AGA AGT | Simon et al., 2004 | 94°C (15’), [94°C(30''), 50°(90''), 72°(2’)]x34, 72°C(4’) |
|  |  | COI R 3014 | TCT AAT GCA TTA ATC TGC CAT ATT A |  |  |
|  | COII | TL2-J-3037 | ATG GCA GAT TAG TGC AAT GG | Austin et al., 2002 | 94°C (5’), [94°C(1’), 45°(1’), 72°(1’)]x30, 65°C(4’) |
|  |  | TK-N-3785 | GTT TAA GAG ACC AGT ACT TG |  |  |
| nuclear | ITS2 | ITS2 F | TGT GAA CTG CAG GAC ACA T | Jenkins et al., 2001 | 94°C (2’), [94°C(30’’), 46°(45’’), 72°(45’)]x30, 72°C(7’) |
|  |  | ITS2 R | GCT TAA ATT TAG GGG GTA GTC |  |  |
|  | Microsat. |  | DeHeer et al., 2005 ; Dronnet et al., 2004 ; Vargo 2000 | | 94°C (5’), [94°C(1’), 55°(1’), 72°(15’’)]x40, 72°C(5’) |


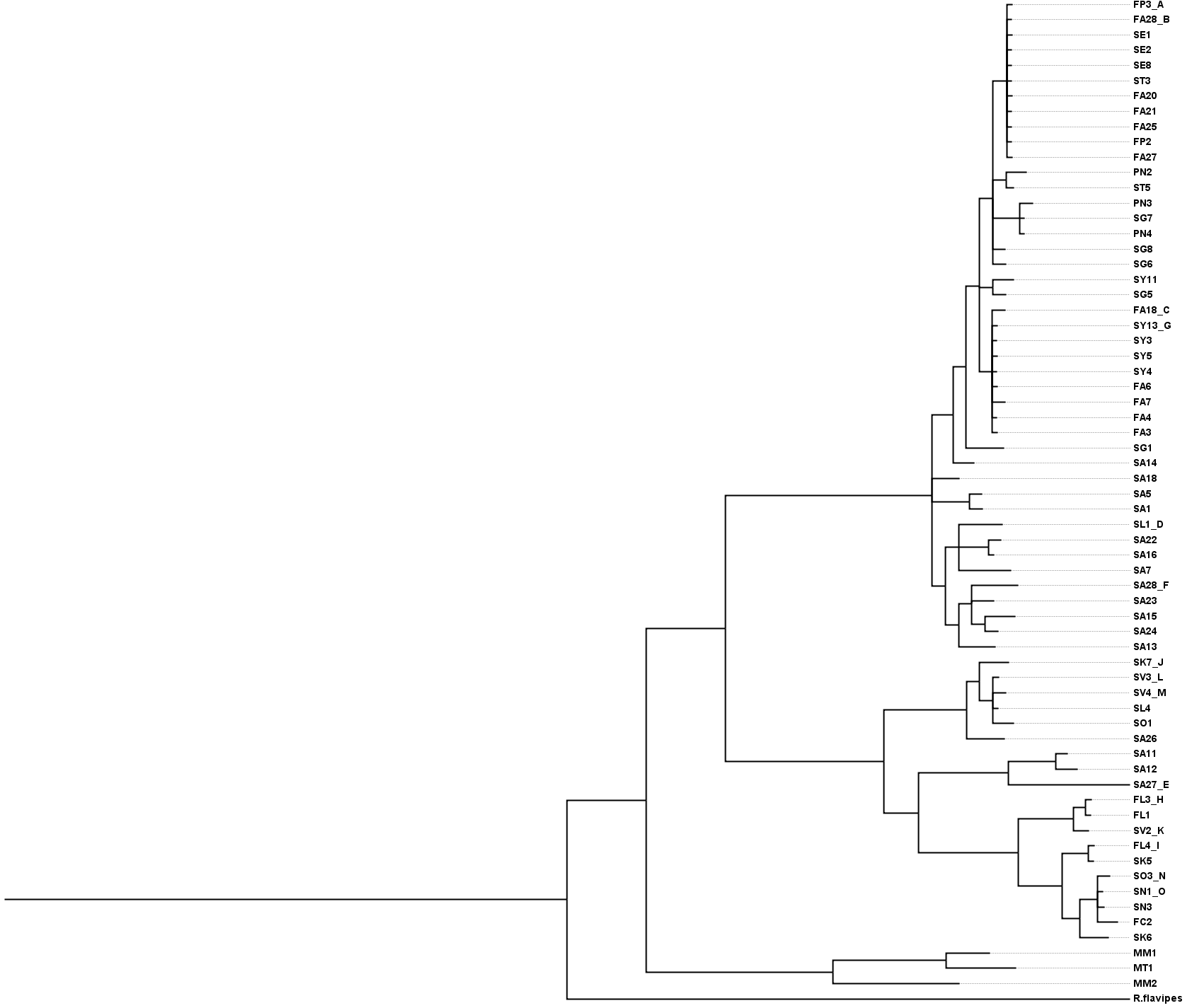
**Figure S1**

**96.7 / 89.8 / 100**

**52.8 / 60.8 / 62.3**

**- / 60 / 98,8**

**100 / 100 / 100**

**100 / 98 / 100**

**100 / 98.4 / 100**

**- / 54.6 / 99.4**

**- / 56.3 / 98.4**

**100 / 97.4 / 100**

**92.8 / 87 / 72.1**

**98.8 / 99.2 / 100**

**57.4 / - / 95,2**

**99.9 / 94.5 / 100**

**94.6 / 86.8 / 100**

**91.6 / 93.2 / 100**

**87.6 / 75.4 / 99.3**

**51.6 / 61.1 / 99.7**

**99.6 / 92.1 / 100**

**99.3 / 97.1 / 100**

**71.4 / 87.8 / 100**

**- / - / 98.4**

**- / - / 98.5**

**- / - / 93.4**

**- / - / 97.1**

**97.3 / 93.4 / 100**

**- / - / 98,3**

**100 / 90.3 / 100**

**- / 87 / 100**

**53.2 / - / 99,6**

**- / - / 99,4**

**73.4 / 60.5 / 99,6**

**93.5 / 80.4 / 100**

**- / - / 99,4**

**- / - / 64,2**

**63.3 / 60.5 / 99,5**

**Outgroup**

**M-3 Moroccan clade**

**M-2 *R. banyulensis*clade**

**M-2b**

**M-2c**

**M-2a**

**M-1 *R. grassei* clade**

**Figure S2**

***R. banyulensis***

***R. grassei***

##
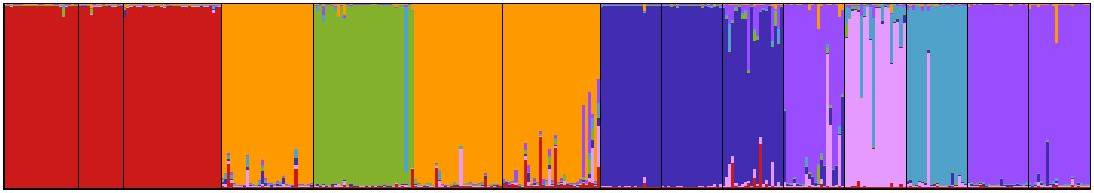

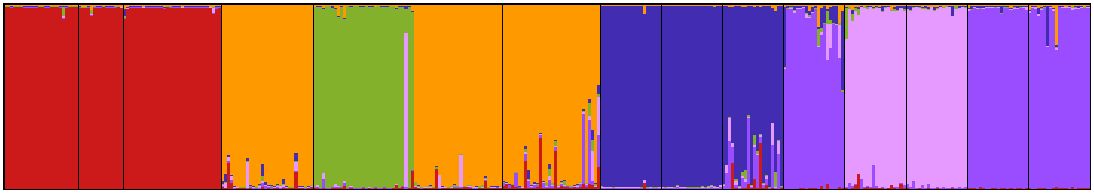

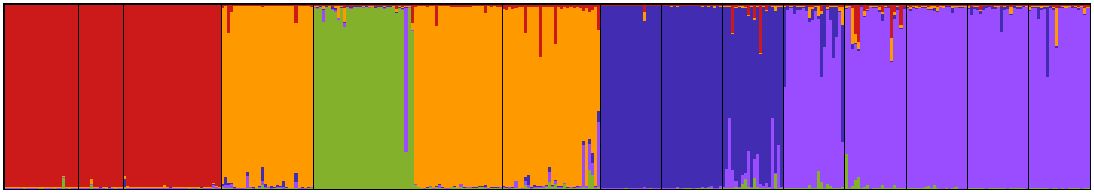

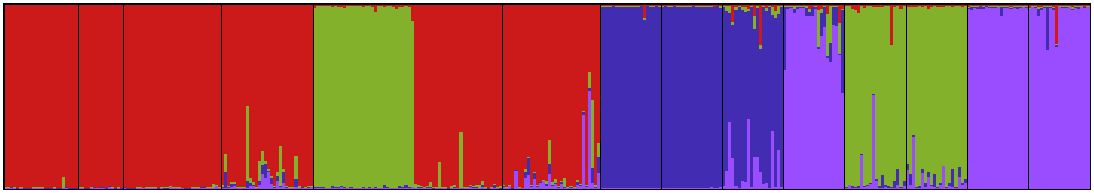

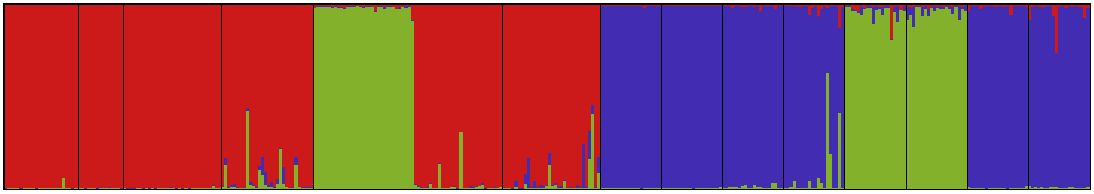

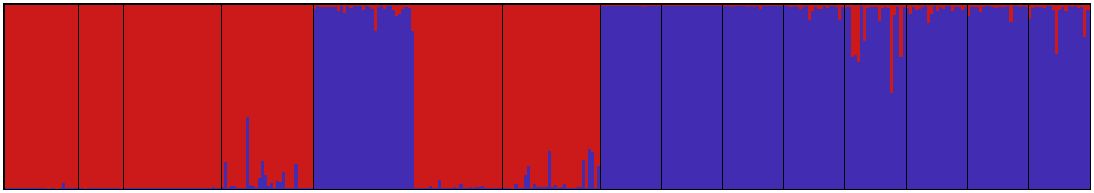


**A B C D E F G H I J K L M N O**

**K=2**

**K=3**

**K=4**

**K=5**

**K=6**

**K=7**

A B C D E F G H I J K L M N O

**Table S2**

| *Lab Code* | Code | Locality (Country, Region, Province) | COI | COII | ITS2 |
| --- | --- | --- | --- | --- | --- |
| *MRI* | MM1 | Ifrane (Morocco, Meknes) | JQ431092 | JQ431042 | KP721558 |
| *XIW* | MM2 | Ajabo (Morocco, Meknes) | JQ431094 | JQ431044 | JQ431133 |
| *MRA* | MT1 | Asilah (Morocco, Tanger) | JQ431093 | JQ431043 | KP721559 |
| *TRF* | SA1 | Tarifa (Spain, Andalucía, Cadiz) | JQ431055 | JQ431005 | JQ431102 |
| *CNF* | SA3 | Conil de la Frontera (Spain, Andalucía, Cadiz) | - | - | JQ431118 |
| *MED* | SA4 | Medina Sidonia (Spain, Andalucía, Cadiz) | - | - | JQ431113 |
| *CHI* | SA5 | Chipiona (Spain, Andalucía, Cadiz) | JQ431047 | JQ430997 | JQ431101 |
| *CAA* | SA7 | Casares (Spain, Andalucía, Malaga) | JQ431046 | JQ430996 | - |
| *COL* | SA8 | El Comenar (Spain, Andalucía, Malaga) | - | - | JQ431121 |
| *MIJ* | SA10 | Mijas (Spain, Andalucía, Malaga) | - | - | AY510538 |
| *SAL* | SA11 | Salares (Spain, Andalucía, Malaga) | JQ431083 | JQ431033 | AY510539 |
| *ATQ* | SA12 | Antequera (Spain, Andalucía, Malaga) | JQ431082 | JQ431032 | AY510540 |
| *CNL* | SA13 | Canales (Spain, Andalucía, Grenada) | JQ431048 | JQ430998 | JQ431122 |
| *PZA* | SA14 | La Peza (Spain, Andalucía, Grenada) | JQ431054 | JQ431004 | - |
| *GDA* | SA15 | El Molinillo (Spain, Andalucía, Grenada) | JQ431050 | JQ431000 | - |
| *PEDR* | SA16 | El Pedroso (Spain, Andalucía, Sevilla) | JQ431053 | JQ431003 | JQ431100 |
| *LORA* | SA18 | Lora del Rio (Spain, Andalucía, Sevilla) | JQ431051 | JQ431001 | - |
| *PAL* | SA19 | Palenciana (Spain, Andalucía, Cordoba) | - | - | JQ431095 |
| *RAB* | SA21 | Rabanales (Spain, Andalucía, Cordoba) | - | - | JQ431117 |
| *CRM* | SA22 | Cerro Muriano (Spain, Andalucía, Cordoba) | JQ431049 | JQ430999 | JQ431116 |
| *ADM* | SA23 | Adamuz (Spain, Andalucía, Cordoba) | JQ431045 | JQ430995 | JQ431120 |
| *MTR* | SA24 | Montoro (Spain, Andalucía, Cordoba) | JQ431052 | JQ431002 | - |
| *CAR* | SA25 | Cardena (Spain, Andalucía, Cordoba) | - | - | JQ431096 |
| *BEL* | SA26 | Belmez (Spain, Andalucía, Jaen) | JQ431086 | JQ431036 | JQ431098 |
| *CIB* | SL4 | Casas Ibanes (Spain, Castilla La Mancha, Albacete) | JQ431084 | JQ431034 | - |
| *MDJ* | SO1 | Mas de Jacinto (Spain, Aragon, Teruel) | JQ431085 | JQ431035 | JQ431132 |
| *TAF* | SN3 | Tafalla (Spain, Navarra, Navarra) | JQ431089 | JQ431039 | JQ431130 |
| *ARV* | SY3 | Arevalo (Spain, Castilla Y León, Avilla) | JQ431056 | JQ431006 | - |
| *VGZ* | SY4 | Veganzones (Spain, Castilla Y León, Segovia) | JQ431059 | JQ431009 | - |
| *CPS* | SY5 | Campaspero (Spain, Castilla Y León, Valladolid) | JQ431057 | JQ431007 | - |
| *LPJ* | SY7 | La Pedreja (Spain, Castilla Y León, Valladolid) |  |  | JQ431097 |
| *CRV* | SY11 | CervatosdiaCueza (Spain, Castilla Y León, Palencia) | JQ431058 | JQ431008 | JQ431115 |
| *OLM* | PN1 | Olmos (Portugal, Norte) | - |  | JQ431107 |
| *SGH* | PN2 | Sanguinhedo (Portugal, Norte) | JQ431067 | JQ431017 | JQ431126 |
| *GOI* | PN3 | Goios (Portugal, Norte) | JQ431063 | JQ431013 | JQ431110 |
| *MOL* | PN4 | Moledo (Portugal, Norte) | JQ431065 | JQ431015 | - |
| *MOS* | SG1 | Mos (Spain, Galicia, Pontevedra) | JQ431066 | JQ431016 | JQ431108 |
| *OLE* | SG4 | Oleiros (Spain, Galicia, Coruña) | - | - | JQ431111 |
| *ALD1* | SG5 | Aldeavella (Spain, Galicia, Coruña) | JQ431060 | JQ431010 | - |
| *ALD2* | SG6 | Aldeavella (Spain, Galicia, Coruña) | JQ431061 | JQ431011 | - |
| *LIR* | SG7 | Lires (Spain, Galicia, Coruña) | JQ431064 | JQ431014 | - |
| *DEV* | SG8 | Devesa (Spain, Galicia, Coruña) | JQ431062 | JQ431012 | - |
| *SEA1* | ST1 | Seares (Spain, Asturias, Oviedo) | - | - | JQ431114 |
| *MEDR* | ST3 | Medredos (Spain, Asturias, Oviedo) | JQ431072 | JQ431022 | - |
| *VILF* | ST4 | Villafria (Spain, Asturias, Oviedo) | - | - | JQ431099 |
| *FIO* | ST5 | Fios (Spain, Asturias, Oviedo) | JQ431069 | JQ431019 | JQ431112 |
| *ARB* | SE1 | Arborteretza (Spain, Euskadi, Bilbao) | JQ431068 | JQ431018 | JQ431109 |
| *GUE* | SE2 | Guernica (Spain, Euskadi, Bilbao) | JQ431070 | JQ431020 | - |
| *LEK* | SE8 | Lekeitio (Spain, Euskadi, Bilbao) | JQ431071 | JQ431021 | JQ431106 |
| *GOR* | SE10 | Goronaeta (Spain, Euskadi, San Sebastian) | - | - | JQ431105 |
| *BOI* | SK5 | SantBoi (Spain, Cataluña, Barcelona) | JQ431087 | JQ431037 | - |
| *CRU* | SK6 | Cruïlles (Spain, Cataluña, Gerona) | JQ431088 | JQ431038 | - |
| *BAY* | FA2 | Bayonne (France, Aquitaine, 64) | - | - | JQ431127 |
| *SPN* | FA3 | St Pée sur Nivelle (France, Aquitaine, 64) | JQ431081 | JQ431031 | - |
| *DMZ* | FA4 | Domezain (France, Aquitaine, 64) | JQ431079 | JQ431029 | - |
| *ATX* | FA6 | Artix (France, Aquitaine, 64) | JQ431073 | JQ431023 | - |
| *AYH* | FA7 | Ayherre (France, Aquitaine, 64) | JQ431074 | JQ431024 | - |
| *ODR* | FA15 | Ondres (France, Aquitaine, 40) | - | - | JQ431125 |
| *BGL* | FA20 | Bouglon (France, Aquitaine, 47) | JQ431075 | JQ431025 | JQ431124 |
| *CAU* | FA21 | Caudecoste (France, Aquitaine, 47) | JQ431076 | JQ431026 | JQ431119 |
| *CHA* | FA25 | La Roche Chalais (France, Aquitaine, 24) | JQ431077 | JQ431027 | - |
| *GSC* | FA27 | Giscos (France, Aquitaine, 3) | JQ431080 | JQ431030 | - |
| *NOG* | FM1 | Nogaro (France, Midi-Pyrénées, 32) | - | - | JQ431103 |
| *PERP* | FL1 | Perpignan (France, Languedoc-Roussillon, 66) | JQ431091 | JQ431041 | JQ431128 |
| *BEZ* | FL2 | Béziers (France, Languedoc-Roussillon, 34) | - | - | JQ431129 |
| *COU* | FP1 | Coubre (France, Poitou-Charentes, 17) | - | - | JQ431104 |
| *CHT* | FP2 | Châtellerault (France, Poitou-Charentes, 86) | JQ431078 | JQ431028 | JQ431123 |
| *MRS* | FC2 | Marseille (France, Provence-Alpes-Côtes d’Azur, 13) | JQ431090 | JQ431040 | - |
| *Lab Code* | Code | Locality (Country, Region, Province) | COI | COII | ITS2 |
| *CAS*  *A*  *B*  *C*  *D*  *E F G H*  *I*  *J*  *K*  *L*  *M*  *N*  *O* | FC3  FP3/A  FA28/B  FA18/C  SL1/D  SA27/E  SA28/F  SY13/G  FL3/H  FL4/I  SK7/J  SV2/K  SV3/L  SV4/M  SO3/N  SN1/O | Cassis (France, Provence-Alpes-Côtes d’Azur, 13)  La Tremblade (France, Poitou-Charentes, 17)  Ychoux (France, Aquitaine, 40)  Pissos (France, Aquitaine, 40)  Ciudad Real (Spain, Castilla La Mancha)  Lucena (Spain, Andalucía, Córdoba)  Guadix (Spain, Andalucía, Grenada)  Cantalejo (Spain, Castilla Y León, Segovia)  Narbonne (France, Languedoc-Roussillon, 11)  Banyuls (France, Languedoc-Roussillon, 66)  Igualada (Spain, Cataluña, Barcelona)  Sant Mateu (Spain, Valencia, Valenciana)  Segorbe (Spain, Valencia, Valenciana)  Alicante (Spain, Valencia, Alicante)  Zuera (Spain, Aragon, Zaragosa)  Estella-Lizarra (Spain, Navarra, Navarra) | -  KP721513  KP721514  KP721515  KP721516  KP721517  KP721518  KP721519  KP721520  KP721521  KP721522  KP721523  KP721524  KP721525  KP721526  KP721527 | -  KP721528  KP721529  KP721530  KP721531  KP721532  KP721533  KP721534  KP721535  KP721536  KP721537  KP721538  KP721539  KP721540  KP721541  KP721542 | JQ431131  KP721543  KP721544  KP721545  KP721546  KP721547  KP721548  KP721549  KP721550  KP721551  KP721552  KP721553  KP721554  KP721555  KP721556  KP721557 |
|  |  |  |  |  |  |
|  |  |  |  |  |  |
